# The Key Elements for Biomolecules to Biomaterials and to Bioapplications

**DOI:** 10.3390/biom12091234

**Published:** 2022-09-04

**Authors:** Deng-Guang Yu, Ping Zhao

**Affiliations:** School of Materials Science and Chemistry, University of Shanghai for Science and Technology, Shanghai 200093, China

Biomolecules, as molecules which have a bio-source or a certain bioapplication, are at present quickening the marching speed for benefiting people’s life and social progress. There are several key elements for their material conversion processes, i.e. their ways from biomolecules to biomaterials and to bioapplications, which are diagrammed in Graphical Abstract. In the sunshine of modern science and technology, these elements will act together to ensure their material transformation route reasonable and efficacious. The present issue “Biomolecule-based composites, hybrids and nanostructures for biomedical applications” is just centered around these elements and thus seven excellent publications have been selected for publication.

New kinds of biomolecules, regardless of whether they are active or inert, are always increasing, with a very limited number of them go into the real commercial products. Both finding new types of biomolecules, and a reasonable selection of the starting biomolecules for preparing biomolecule-based functional materials, are important. In this issue, Kuczumow et al. found two types of biological apatites in molar sheep and horse teeth, identified two kinds of ion-exchanges responsible for formation of peculiar apatites, and disclosed that various combinations of main and minor elements led to new versions of biological apatites [1]. Wang et al. reported that a special biomolecule, E7-QK peptide, is able to excellently endow the collagen/hydroxyapatite composites fine angiogenic properties [2].

Both inert and active biomolecules are frequently explored to prepare functional biomaterials, with most of them presenting in a composite or hybrid format and few of them in a pure state. Thus, the reasonable design of the biomaterials (such as outer shape, inner structure, and components and compositions) and the applied techniques are vital. In this issue, Li et al. reported a special shape and also a special structure, i.e. Janus beads-on-a-string, for furnishing a biphasic drug release profile [3]. The selected inert biomolecule ethyl cellulose as matrix, the beads-on-a-string shape, and the inner Janus two-chamber structure have acted together to ensure the desired drug-controlled release behaviors. A side-by-side electrospinning process was exploited for the preparation. In another job, Xu et al. reported a modified tri-axial electrospinning, which was utilized to prepare a core–sheath nanostructure of cellulose acetate. The fibers were verified to be able to release the loaded drug in an improved sustained manner [4]. These biomolecule-based structures are general hybrid materials, but nanocomposites in terms of each compartment within the multiple-chamber structures. This has also been demonstrated by other core–sheath [5], tri-section Janus [6], tri-layer core–sheath nanostructures [7], and fiber-casting film hybrid structures [8]. Furthermore, a fine review about chitosan-based composites is published in this issue. Their recent fabrications and implant and dentist applications are excellently concluded [9]. Biomolecules can be organic, or inorganic, can be from nature or chemical synthetic. A most recent trend for constructing new biomolecules of biomolecule-based composites is to the combination of organic and inorganic elements. An example is the metal-organic frameworks [10]. In this issue, Salama et al. summarized the composites from the combination of cellulose and silver nanoparticles, their fabrication methods, their unique properties, and their potential applications in a wide variety of fields [11].

The aim of material conversion from biomolecules to biomaterials is to endow them a certain functional performance. This is the most fundamental relationship between man and nature. In general, there should no application limitations about the biomolecule-based composites, hybrids and nanostructures. They can be exploited by all scientific applied fields for healthy life and strong body, such as agriculture, industry, forestry, health and hydrogen, disease treatment, epidemic prevention, energy, environment, and so on. For example, Jiang et al. recently reported a zein-based film for active food packaging, which showed good antibacterial performance [12]. Anyway, the mainstream of bioapplications for biomolecules should be medical applications, such as drug delivery [13], diagnosis [14], scaffolds [15], wound dressing [16], medical engineering (sutures) [17], and herbal extraction and medicines [18]. Here, Hwang et al. reported a new type of biomolecule-based composites containing magnetic nanoparticle, resorufin β-d-glucopyranoside and β-glucosidase. Compared with the commercial lateral flow assay diagnostic kit, their products exhibited four-fold sensitive and more accurate, could provide an optical and electrochemical dual-modal detection on the Foot-and-mouth disease virus serotypes O and A [19]. In general, based on the combinations of biomolecules and modern technologies, there should be more and more possibilities for amplifying all kinds of biomedical applications, particularly drug delivery [20] and tissue engineering [21].

For the biomedical applications of functional materials, the systematic analyses and characterizations are important. These analysis techniques, on one hand, can ensure the materials properties for the designed functional applications, providing information for updating the materials. On the other hand, they help to disclose the process-property-performance relationship, which in turn can deepen and broaden the related knowledge and make the ways from biomolecules to biomaterials and to biomedical applications more reproducible and durable.

In summary, through a manner of peeping through a tube at a leopard, this Special Issue showed some examples of biomolecule-based functional biomaterials and some of the suited ways from the biomolecules to biomaterials, and to biomedical applications. It can be argued that the biomolecule-based composites, hybrids and nanostructures will bring out more and more commercial products, broaden the related knowledge, and provide solutions for some key challenges of biomedical applications in future.
